# Integration of community-based testing data into national HIV surveillance in Poland, Serbia and Slovakia within the framework of INTEGRATE project

**DOI:** 10.1186/s12879-021-06498-6

**Published:** 2021-09-13

**Authors:** Laura Fernàndez-López, Sladjana Baros, Marta Niedźwiedzka-Stadnik, Danica Valkovičová Staneková, Magdalena Rosińska, Danijela Simic, Verica Jovanoic, Monika Hábeková, Mária Takáčová, Iwona Wawer, Piotr Wysocki, Anna Conway, Irena Klavs, Jordi Casabona

**Affiliations:** 1 Centre of Epidemiological Studies of HIV/AIDS and STI of Catalonia (CEEISCAT), Health Department, Generalitat de Catalunya, Badalona, Spain; 2 Institute for Health Science Research Germans Trias i Pujol (IGTP), Badalona, Spain; 3 CIBER Epidemiologia y Salud Publica (CIBERESP), Madrid, Spain; 4 Institute of Public Health of Serbia, Dr Milan Jovanovic Batut”, Belgrade, Serbia; 5 National Institute of Public Health – National Institute of Hygiene (NIPH-NIH), Warsaw, Poland; 6 NRC for HIV/AIDS Prevention, Slovak Medical University, Bratislava, Slovakia; 7 National AIDS Centre (NAC), The agency of the Ministry of Health, Warsaw, Poland; 8 National Institute of Public Health, Ljubljana, Slovenia; 9 Department of Paediatrics, Obstetrics and Gynecology and Preventive Medicine, Univ. Autonoma de Barcelona, Badalona, Spain

**Keywords:** HIV infection, Sexually transmitted infections, Hepatitis, Testing, Community health services, Surveillance, Monitoring and evaluation

## Abstract

**Background:**

Community-based voluntary counselling and testing contributes to early HIV diagnoses among key populations. Testing data from such decentralized services is however often not standardized nor linked to national surveillance systems. This study aimed to support the integration of community testing data into respective national surveillance and monitoring and evaluation systems for those infections. We present results from three national pilots, focused on improved data collection and transfer.

**Methods:**

Within the Joint Action INTEGRATE different pilot activities were planned and implemented according to the local context. In Slovakia, standardised data collection tools were implemented in three community testing services. The data generated was used to calculate the proposed indicators. In Poland, positive test results from the community testing database were linked to the national case-based surveillance database using confirmatory test number, to improve the completeness of behavioural data in the national database. In Serbia, voluntary counselling and testing forms were improved enabling identification of community-based testing. A system to generate unique client identifiers was initiated in the National registry of HIV cases to monitor linkage to care.

**Results:**

All three sites were able to estimate most of the agreed indicators. In Slovakia during the study period 675 people were tested for HIV, 410 for hepatitis C and 457 for syphilis, with reactivity rates of 0.4, 2.5 and 1.8%, respectively. For HIV, 66.7% of reactive cases were confirmed and linked to care. In Poland, 28.9% of the community testing sites’ records were linked to the national surveillance database (and accounted for 14.3% of all new diagnoses registered here during 2017–2018). Reactivity rate ranged between 1.9% and 2.1%. In Serbia, 80 persons were tested at community sites, from which two had a reactive HIV test result. By linking unique client identifiers from voluntary counselling and testing and National Registry of HIV cases databases, linkage to care within a two-month period was observed for one of two people with reactive HIV test result.

**Conclusions:**

Pilot activities in the three countries demonstrate that integration of community-based testing data into surveillance systems is feasible and can help improve national surveillance data by providing key information.

## Background

An effective national testing strategy, including a well-functioning monitoring and evaluation (M&E) framework, is critical in responding to HIV infection, other sexually transmitted infections (STIs), hepatitis B and hepatitis C infections [1, 2].

Community-based voluntary counselling and testing (CBVCT) has been shown to contribute to early diagnosis of new HIV cases, especially among key populations [3–6]. CBVCT services can reach key populations at higher risk of HIV infection and STIs such as men who have sex with men (MSM), sex workers, people who inject drugs and migrants. However, such services are often provided by non-governmental organisations and decentralized, which may pose challenges to implement standardized monitoring systems and link testing data to national surveillance systems.

In the ECDC’s “Monitoring implementation of the Dublin Declaration on partnership to fight HIV/AIDS in Europe and Central Asia in 2018” [6], several countries reported community-based HIV testing delivered by a medical provider or by a lay-provider. Only some of those countries were able to report the number of tests performed and the positivity rate in community-settings. In addition, the CBVCT services are often designed to offer integrated testing for several infections, relevant in the served population. One challenge to integrate these CBVCT data into national surveillance and M&E systems relates to the fact that quite often national surveillance systems in Europe are based on case reporting and are organized around specific diseases. In some countries there are also some legal issues related with CBVCT activities which further impose barriers for monitoring them [6, 7]. Community-based testing (not only for HIV but also for STIs and viral hepatitis) is growing in the European Union (EU), and this type of testing makes a vitally important contribution to early diagnosis, especially for key populations.. For a coherent and informed national response to HIV, STIS, hepatitis B and hepatitis C virus, community-based testing should be included in every country’s surveillance and M&E framework[2].

In recent years there has been an increasing interest in CBVCT activity monitoring from some initiatives and institutions like the ECDC [1]. Two European projects (HIV-COBATEST and EURO HIV EDAT) have previously developed and recommended a group of core indicators [8, 9] to monitor and evaluate HIV testing in the community, of which some indicators have been incorporated into the Dublin Declaration HIV service monitoring process.

The “Joint Action on integrating prevention, testing and linkage to care strategies across HIV, viral hepatitis, tuberculosis and STIs in Europe” (INTEGRATE)[10] has the overall objective to increase early diagnosis and linkage to prevention and care not only for HIV and viral hepatitis, but also for tuberculosis and STIs in EU Member States by 2020. One of the objectives was to support the integration of testing and linkage to care data obtained at CBVCT sites into national surveillance and M&E systems for HIV, STIs, hepatitis B and hepatitis C virus. To assess the feasibility of integrating a set of proposed CBVCT indicators into national surveillance and M&E systems pilot studies were conducted in 6 countries (Estonia, Poland, Serbia, Slovenia, Slovakia and Spain).

This article presents results from three national pilots, focused on improved data collection and transfer: Serbia’s integration of a linkage to care indicator; Poland’s integration of CVBCT data into national case-based surveillance (NCBS); and Slovakia’s use of standardized data collection tools in CBVCT services. The focus of the three other pilots was slightly different, (more centred on HCV indicators in Spain; in an agreement on how to share the indicators between the CBVCT service and the National Public health Institute in Slovenia; and in a reinforcement of CBVCT in Estonia) and their results are not presented in this article.

## Methods

### Pilot countries and indicators

Representatives from public health bodies and community organisations from six participating countries (Estonia, Poland, Serbia, Slovenia, Slovakia and Spain) convened in November 2018 to agree a core set of indicators to collect during pilot activities (Table 1). In this article we present the pilot results from three of those countries focused on improved data collection and transfer.

**Table 1:** Consensus CBVCT M&E core indicators to be integrated into national surveillance and M&E systems*

Minimum set for HIV, hepatitis B and C, STIs:
Number of tests Number of clients tested Reactivity rate of tests Reactivity rate of clients Positivity or active infection rate of tests Positivity or active infection rate of clients Proportion of clients with a positive result linked to care Proportion of all new diagnoses with first reactive test at CBVCT
Extended set for HIV, HCV, STIs:
Proportion of clients who reported to have been previously tested Proportion of clients who reported to have been tested during preceding 12 months Proportion of clients with reactive screening test result tested with confirmatory tests Proportion of clients tested at specific venues: office, outreach, self sampling,…
Extended set only for syphilis and HCV:
Proportion of clients who reported to have been previously diagnosed​

*As well as the overall estimate, each indicator should be disaggregated by key population, gender and age

*CBVCT* Community-Based Voluntary Counselling and Testing

### Needs assessment

Prior to implementing the pilots, a needs assessment was conducted in each participating country regarding the current status of integration of HIV, STIs and viral hepatitis testing and linkage to care data from CBVCT services into national surveillance and M&E systems. The pilot activities in each country were planned according the needs assessment and identified challenges.

In Slovakia, there are four CBVCT services performing HIV testing, one of them, a checkpoint, is targeting MSM. Only one of the CBVCT services was collecting testing data with a questionnaire, and there was no standardized CBVCT data base. In the case of a reactive HIV result, CBVCT services cooperate with the National Reference Centre for HIV/AIDS prevention for the confirmatory testing and the National Reference Centre sends a report to the Institute of Public Health who, in turn, submits the result to the Epidemiological Information System. There was no legislation regulating for CBVCT services’ data integration with the national systems.

In Poland, the National Aids Centre operates a network of approximately 30 CBVCT services offering HIV testing to 30,000 clients annually, mainly from key populations. The network relies strongly on close cooperation between the governmental system and NGOs. Since 2016, the National Aids Centre has used an electronic data collection system to collect data from CBVCT services. In addition to HIV, some CBVCT services offer testing for STIs and Hepatitis C, but as the mandate of the National Aids Centre is only HIV, the legal framework did not allow data to be collected on these diseases. Collected data was analysed by the National Aids Centre and shared with the National Institute of Public Health – National Institute of Hygiene that is responsible for the national surveillance. However, the systems’ incompatibility and data protection issues hindered full use of this data. The CBVCT database contains anonymous behavioural data at the individual level for all clients, together with laboratory confirmatory test results. Laboratories are obliged to report positive confirmatory results to the national case-based surveillance, including anonymous results from CBVCT samples, but as they are not obliged to gather information on patients’ behaviours, there are a lot of missing data about these variables. So integration of the CBVCT database with national case-based surveillance could improve surveillance data, by adding behavioural information. In addition, linkage to care could be assessed by identifying cases for which a clinical report is received.

In Serbia seven NGOs/civil society organisations are providing CBVCT services for HIV and occasionally for HCV (HBV and syphilis were offered on one or two occasions in the last eight years) in cooperation with district Institutes of Public Health. Official CBVCT data for each client was included in the national voluntary, counselling and testing (VCT) database (administered by the district Institutes of Public Health), but was not identified as such, so it was not possible to see how many people were tested in the community. The National HIV database doesn’t include data from VCT database at all (basic demographic data, risk behaviour data, HIV testing results, previous HIV testing, data on other STDs testing, etc.), just whether clients with a unique identifier were reached with different HIV preventive services. Data from National HIV database includes limited information so can’t be used for CBVCT monitoring. Data on confirmatory testing can’t be included in CBVCT reporting due to legal restrictions to protect client privacy, so it’s not possible to have linkage to care data.

### Pilot activities

Table 2 summarizes the specific aims, the indicators specifically addressed and CBVCT services participating in each pilot.

**Table 2:** Specific aims, the indicators that could be already assessed before the pilot, the indicators specifically addressed in the pilot and CBVCT services participating in each pilot

Pilot country	Specific aims for the pilot	Indicators that could be already assessed before the pilot	Indicators specifically addressed	Number of CBVCT services participating in the pilot out of the total number of CBVCT services in the country
Slovakia	To implement standardised data collection tools in the CBVCT services, as most of them are not using a questionnaire	For HIV, Syphilis and HCV: Number of testsReactivity rate of tests	For HIV, Syphilis and HCV: Number of clients tested Reactivity rate of clients For HIV: Proportion of clients with a positive result linked to care	3 out of 4
Poland	Linkage of positive test results from community testing database to the national casebased surveillance database using confirmatory test number To include testing for Syphilis and HCV in the electronic data collection system for CBVCT services	For HIV: Number of tests Reactivity rate of tests	For HIV: Proportion of clients with a positive result linked to care	30 out of 30 form the national VCT network *(There are a few more testing sites for PWID, operated by harm reduction centres)*
Serbia	Improvement of voluntary counselling and testing forms to enable identification of communitybased testing use of unique client identifier to monitor linkage to care	For HIV: Number of tests Reactivity rate of test *(both indicators had to be obtained by manual counting)* Number of clients tested Reactivity rate of clients *(for both indicators data was available but never used at national level)*	For HIV: Number of tests Number of clients tested Reactivity rate of clients Proportion of clients with a positive result linked to care Proportion of clients who reported to have been previously tested Proportion of clients who reported to have been tested during preceding 12 months Proportion of clients tested at specific venues: office, outreach, self sampling,…	2 out of 7 *(at mid 2019 there were 7 CBVCT services, but the this number is not fixed, with tendency to increase)*

*CBVCT* CommunityBased Voluntary Counselling and Testing

In Slovakia, the pilot consisted of the implementation of an online standardised data collection instrument (from the COBATEST network [11]) in the CBVCT services. In addition, there were negotiations with the National AIDS Committee and epidemiologists from the Ministry of Health and the Institute of Public Health to integrate a minimum set of CBVCT indicators into the national epidemiological information system.

In Poland, pilot activities aimed to extend the National Aids centre’s mandate beyond HIV/AIDS to STI prevention; to integrate STIs and viral hepatitis into the electronic VCT database; to share data from the National Aids Centre CBVCT database with National Institute of Public Health – National Institute of Hygiene, to allow linkage of positive CBVCT samples by confirmatory test number to improve completeness of behavioural data in national case-based surveillance. The pilot intended to assess feasibility of matching cases by existing variables (i.e. confirmatory test number), to understand to what extent data integration could improve the completeness of exposure category variable in national case-based surveillance dataset. The pilot also intended to assess if linkage to care of the CBVCT clients who were diagnosed with HIV could be calculated through surveillance data.

In Serbia pilot activities focused on improving electronic and paper VCT forms and the VCT database, by including new questions and variables, allowing identification of CBVCT testing, and using a unique client identifier (UCI). At the same time, the National registry of HIV cases, with data on HIV positive persons and administered by national Institute of Public Health, implemented a system to assign a VCT UCI for each reported HIV diagnosed case, in order to monitor linkage to care by matching cases across VCT and National registry of HIV cases databases.

### Study period

The pilot activities were implemented between 1st January 2019 and 31st June 2019. Some pilot activities were extended to 31st August 2019. The time period used for estimating the indicators differed in each country: 6 months in Slovakia (03/2019–08/2019); 2 months in Serbia (06/2019–07/2019); and two years of retrospective data were used in Poland, (01/2017–12/2018).

## Results

### Pilot results

The three pilot countries were able to estimate some of the CBVCT indicators and integrate them into their surveillance and M&E systems. The results of the main indicators are summarised in Table 3.

**Table 3:** Results overview in each pilot country

Country	Data collection/analysed period	People tested in CBVCT services	Reactivity rate	Linkage to care	Linkage to HIV registry cases
Slovakia	6 months (03/2019–08/2019)	HIV: 675 HCV: 410 Syphilis: 457	HIV: 0.4% (3/675) HCV: 2.4% (10/410) Syphilis: 1.8% (8/457)	HIV: 66.7% (2/3) HCV: Not known Syphilis: Not known	66.7% (2/3)
Serbia	2 months (06/2019–07/2019)	HIV: 80	2.5% (2/80)	50% (1/2)	50% (1/2)
Poland	2 years 2017–2018	HIV (tests): 2017: 29,353 2018: 28,348	2017: 2.1% (616/29353) 2018: 1.9% (539/28348)	2017: 36.4% (82/225) 2018: 43.9% (65/148)	2017: 38.3%(225/588) 2018: 31.8% (148/465)

*CBVCT* Community-Based Voluntary Counselling and Testing

In Slovakia, three CBVCT services participated in the pilot, performing tests for HIV, syphilis and HCV. Two services used the COBATEST Network online data collection tools, and the third service, who was already using their own online questionnaire, agreed to share the necessary data. The use of a standardised data collection tool for CBVCT services ensured the CBVCT indicators could be easily estimated. During the study period, 675 people were tested for HIV in CBVCT services, 410 for HCV and 457 for syphilis, with reactivity rates of 0.4% (n = 3), 2.5% (n = 10) and 1.8% (n = 8), respectively (Table 3). For HIV 66.7% (n = 2) of the reactive cases were confirmed and linked to care. All positive cases were also linked with Slovakia’s epidemiological information system. Linkage to care for syphilis and HCV reactive cases is unknown, as depends on different circuits which were not actively involved in the pilot. Core indicators were not integrated into the Epidemiological Information System as the system only collects information on positive cases and not on all tests performed.

In Poland, the National Aids Centre CBVCT database was shared with National Institute of Public Health—National Institute of Hygiene, allowing retrospective linkage of CBVCT records with the national case-based surveillance database. Of all CBVCT records, 35.4% (373/1053) were linked to the national case-based surveillance database through the Western Blot number (cases already reported in the national case-based surveillance by laboratories), corresponding to 14.3% (373/2615) of all new diagnoses in this database during the analysed period. The rest of CBVCT records could not be linked due to missing Western Blot numbers or cases not reported by laboratories. Using CBVCT data, transmission route and nationality could be completed for 72.9% (768/1053) of linked cases in the national case-based surveillance database. Additionally, information on prior diagnoses from the CBVCT database was used to remove duplicates in the national case-based surveillance database. The reactivity rate ranged from 1.9% (539/28348) in 2018 to 2.1% (616/29353) in 2017. The proportion of cases with a positive confirmatory test that were linked to care in the period 2017–2018 was 39.4% (147/373)(according to the cases linked to national case-based surveillance database). In August 2019 National Aids centre’s mandate was extended beyond HIV/AIDS to STI prevention, facilitating in the future the collection of routine testing data for STIs, HCV and HBV through the CBVCT online data collection system. Currently, the National Aids Centre is examining the possibility of introducing integrated testing for HIV and other STIs in all VCTs in Poland bearing in mind all legal, logistical and technical aspects of the project.

In Serbia, improvements in the VCT database were implemented to measure the contribution of CBVCT services in the total number of new diagnoses. During the data collection period of two months, 80 people were tested at CBVCT sites, of which two had a reactive HIV test result. By matching UCIs from the VCT database, where the CBVCT data was entered, to the National registry of HIV cases database, linkage to care within the two month period was observed for one of two people with reactive HIV test result. The second person with a reactive HIV test result was not found in the National registry of HIV cases database, despite reporting that he was aware of his diagnosis and on treatment.

### Barriers and facilitators

The pilots identified several barriers and facilitators for the integration of CBVCT testing and linkage to care data into national surveillance and M&E systems (Table 4).

**Table 4:** Barriers and facilitators for CBVCT data integration into surveillance and M&E systems

Country pilot		Barriers	Facilitators
Slovakia	Information Technology	Lack of standardized data collection tools Technical problems integrating CBVCT indicators into Epidemiological Information System	Existence of free standardized online data collection instruments
	Legal issues	Due to lack of public health insurance, some clients tested in CBVCT services are not able to receive healthcare. People who use drugs are not allowed to receive HCV treatment	
	Inter organizational relations		Good relations among the different stakeholders (CBVCT service, National Reference Centre for HIV/AIDS prevention, Public Health institute, Ministry of Health)
Poland	Information Technology	Lack of common UCI across the CBVCT database and national case-based surveillance database	Existence of an online data collection tool and a centralized electronic CBVCT database
	Legal issues	Limited mandate of National Aids Centre (dedicated only to HIV prevention) prevented testing for other STIs in VCT services financed by the National Aids centre Legislation for linkage of different databases and data ownership Lack of unique identifier in CBVCT system Due to lack of insurance some clients who were tested in VCT cannot be linked to care and receive ART as well as treatment for hepatitis	Some CBVCT services are already performing testing for other STIs and HCV Data protection and full anonymity for clients can be guaranteed through linkage of anonymous data The extension of the National Aids centre’s mandate beyond HIV/AIDS to STIs prevention, since August 2019 extend the National Aids centre’s mandate beyond HIV/AIDS to STI prevention
	Inter organizational relations		INTEGRATE project facilitated the cooperation among different stakeholders, mainly among National Aids Centre and National Institute of Public Health – National Institute of Hygiene
Serbia	Information Technology	Data for building UCI in the National registry of HIV cases have slightly different structure than the predefined national UCI. Extra work is required to manually change the UCI generated from the register	The Center for Informatics and Biostatistics, as a part of the Serbian Institute of Public Health and part of the team which implements INTEGRATE, was committed to making the required changes to the databases
	Legal issues	All changes in VCT instruments have to be formalized, which asks for changes in existing regulation	Recommendation by Ministry of Health to guide collaboration between NGOs and regional/district Institutes of Public Health in order to implement CBVCT The official agreement between NGOs and health institutions facilitates implementation of CBVCT in line with legal requirements, and has been evaluated by NGOs as very useful
	Inter organizational relations	Prior to INTEGRATE, ccooperation among NGOs and health institutions was occasional and not formally focused on reaching key populations	Ministry of Health was supportive in the process of implementing the pilot, as well as regional/district Institutes of Public Health s and other health institutions and NGOs reached with the pilot

*CBVCT* Community-Based Voluntary Counselling and Testing, *UCI* Unique Client Identifier, *VCT* Voluntary Counselling and Testing

In Slovakia, the main barriers were the lack of standardized data collection tools; technical problems for integration of CBVCT data into Epidemiological Information System; legal issues related to access to healthcare for people without public health insurance and the fact that HCV treatment is not provided to people who use drugs. Nevertheless, data integration was facilitated by good relations among the different stakeholders and the availability of a free, standardized data collection tool (the COBATEST Network tool [11]).

In Poland, the main difficulty faced was the limited mandate of the National Aids Centre (only HIV prevention) that prevented integrated testing for other STIs in VCTs financed by the National Aids centre, even in terms of data collection if sites performed integrated testing through different funding. The Joint Action INTEGRATE contributed to the extension of the National Aids Centre mandate to encompass the prevention of other STIs in August 2019. This development is a step towards future centrally funded, systematic integrated testing for several infections in VCTs. With respect to the core CBVCT indicators, the difficulty identified is that CBVCT does not use unique identifier, so multiple results of a single person cannot be linked. This problem propagates also to the national case-based surveillance, as the cases diagnosed in CBVCT are reported anonymously by the labs. The pilot showed that integration of CBVCT and surveillance datasets is possible, although more work would be necessary to ensure the common, but not unique, identifier—the test number—is properly completed. More importantly, there were data protection issues which presented a barrier to integrating data from different sources, particularly with regards to guaranteeing full anonymity.

In Serbia, the main barriers faced were legal issues related to changing the VCT data collection instruments, and technical issues related to the modifications in the VCT database and to the implementation of the same UCI across the VCT and National registry of HIV cases databases. Technical issues were successfully tackled, since the Center for Informatics and Biostatistics from Institute of Public Health was committed to support implementation of changes in both databases. Future integration of piloted changes in the VCT data collection instruments will require changes in the regulation in which VCT instruments and procedures are defined. Prior to the pilot, cooperation among NGOs and health institutions was occasional and broadly formalized for cooperation in all activities and largely did not focus on reaching key populations. During the pilot, the official model of agreement between NGOs and health institutions was developed, focusing on CBVCT implementation (roles and responsibilities of each party). Key stakeholders (including the Ministry of health, regional/district Institutes of Public Health, other health institutions and NGOs involved in the pilot) were supportive in the implementation phase.

## Discussion

Pilot activities in three European countries, demonstrated that integration of CBVCT testing and linkage to care data into national surveillance and M&E systems is feasible and can help improve surveillance and M&E data by providing key information.

Prior to the pilots, some countries had already identified the need to integrate CBVCT data into national surveillance. Participation in the EU co-funded Joint Action INTEGRATE [10] offered the funding and international interest to ensure participants could free up time to dedicate to the pilot activities and the international interest motivated national commitment to change.

Firstly, good communication between all stakeholders, but particularly between state and community organisations, will optimise the integration process and avoid duplication of tasks. We found that CBVCT services were often already collecting the data necessary to construct the set of CBVCT M&E indicators and only the processes of data transfer needed to be improved. Such was the case in Poland, where the pilot allowed investigating the technical and legal possibilities of data sharing and integration. In some cases the pilot activities offered a chance for CBVCT services to build their capacity. Burden on CBVCT services was reduced by using free, user-friendly tools like the COBATEST Network data collection tool [11] or other developed within national surveillance and M&E systems, which facilitated the collection of harmonised data from different sources. Linkage to care pathways were also facilitated by the good relationships between some CBVCT services and the healthcare institutions.

Secondly, the scaling up of testing activity must be coupled with universal access to healthcare. The pilot activities shed light on the legal barriers in some countries for key populations seeking or accessing testing or treatment. This is evident in several ways. Some countries still have restrictions on testing by lay providers and testing in non-medical settings which prevents the development of CBVCT services [6]. Clients with a positive test face barriers to accessing treatment due to restrictions which discriminate against key populations (such Slovakia where people who use drugs are prevented from accessing HCV treatment) or because treatment is too expensive or not refunded for individuals without health care insurance [12].

Thirdly, countries should evaluate their existing infrastructure to understand how it can be adapted to integrate CBVCT data at minimal cost to the governmental structures and the CBVCT services. In most cases the pilot activity presented an extra burden for already-stretched CBVCT services. CBVCT services were compensated during the pilots but with the end of the Joint Action INTEGRATE, the sustainability of the pilot actions will be an issue. For several CBVCT services, the lack of access to tests, lack of funds, availability of appropriate training for lay providers, legal barriers for CBVCT implementation by lay providers, and burdensome data entry tasks will be barriers to sustaining the achievements of the pilots in the long term. CBVCT services need support and commitment from public health bodies, to ensure testing and integration of testing data can be maintained over the long term.

Fourthly, CBVCT data integration should be balanced with the need to maintain client anonymity to increase testing uptake. The use of a UCI that allows the linkage of health records from various facilities is highly recommended [13]. Using UCIs assists in monitoring the progress of individuals through the continuum of HIV prevention and care [13]. In some cases, CBVCT services noted that the use of a unique identifier to monitor linkage to care conflicts with the offer of anonymous testing. Nevertheless, there are several options to use a UCI while maintaining anonymity. For example, services can decide not to collect identifiable information, so if a data breach occurs the database cannot be used to identify individuals receiving services [13, 14]. Poland have historically offered anonymous testing in CBVCT services but in the coming months they plan to begin using a UCI in order to allow follow-up of people diagnosed with HIV or STIs. During the pilot, Serbia started using the same UCI across the VCT and National registry of HIV cases databases to match positive cases and improve monitoring of linkage to care.

One of the big achievements of the pilot in Poland was highlighting the need for integrated HIV and STI testing, so hopefully integration of data on testing for different infections will become feasible. Integrating CBVCT data into national surveillance and M&E systems is very important, and will improve some gaps in national surveillance and M&E systems. The pilot in Serbia had significant impact on its national system of M&E and surveillance. It helped to start process of revision of instruments for measurement of its national HIV response in the field of CBVCT. Moreover, the revision also initiated discussion related to legal grounds for CBVCT, helping to defining the legal framework for conducting CBVCT by NGOs and for their reporting on CBVCT activities. The pilot has been the first step in the process of strengthening the integration of CBVCT data into national M&E and surveillance system. In Slovakia, the pilot has been beneficial for CBVCT services, helping them to use the COBATEST Network free data collection tools to collect standardized data. The results of the pilot could help stakeholders to be interested in CBVCT services’ activities, supporting them, and will encourage them to use the minimal set of indicators in the national surveillance system.

There were several limitations to this study. Countries implemented different pilots that responded to their country’s context, therefore the results are not comparable across countries. The timeframe for the pilots differed between countries as some needed to implement structural changes that required more time. We have reported on the achievements of the pilots so far, but the sustainability of each pilot will become evident in the longer term. In spite of these limitations the pilots have identified barriers and facilitators to integrating CBVCT data into national surveillance systems, which will undoubtedly be of use to other countries wishing to do the same.

## Conclusions

Integrating CBVCT data into surveillance and M&E systems can help to determine the contribution of CBVCT to diagnosis of HIV, STIs and viral hepatitis in the country and allow the comparison of data between CBVCT services and between countries.

The integration process differed by pilot country, responding to the specific needs of the country and identifying facilitators and barriers. In Slovakia, the pilot focused on the use of standardised data collection tools to facilitate estimation of CBVCT M&E indicators. In Serbia, implementation of a common UCI based partially on personal data has proved feasible and useful to monitor linkage to care for people with a reactive HIV test result in CBVCT services. In Poland, the surveillance database was enriched with data from CBVCT database while retaining the anonymity of clients tested in CBVCT services. In spite of the differences between countries, we believe the lessons learned from these pilots will be applicable for other countries preparing to integrate their CBVCT data.

The pilot activities have been useful to put the topic of integration of the community-based testing data into national surveillance and M&E systems on each country’s agenda.

Using the pilot activities, the pilot partners reached a consensus on a set of general recommendations for the integration of community-based testing data into national surveillance and M&E systems in other countries (cross-reference to the other article in the supplement: “Recommendations for collection and integration of community-based testing and linkage to care data into national surveillance, monitoring and evaluation systems for HIV, viral hepatitis and sexually transmitted infections; Results from the INTEGRATE Joint Action.”).

## Data Availability

Data sharing is not applicable to this article as no datasets were generated or analysed during the current study.

## Abbreviations

CBVCT: Community-based voluntary counselling and testing
EU: European Union
MSM: Men who have sex with men
M&E: Monitoring and Evaluation
NCBS: National case-based surveillance
NRHC: National registry of HIV cases
STIs: Sexually transmitted infections
UCI: Unique client identifier
VCT: Voluntary, counselling and testing
